# Starvation Survival and Biofilm Formation under Subminimum Inhibitory Concentration of QAMs

**DOI:** 10.1155/2021/8461245

**Published:** 2021-01-14

**Authors:** Sanjay Kumar Tiwari, Suping Wang, Yannan Huang, Xuedong Zhou, Hockin H. K. Xu, Biao Ren, Xian Peng, Yan Xiao, Mingyun Li, Lei Cheng

**Affiliations:** ^1^State Key Laboratory of Oral Diseases & National Clinical Research Center for Oral Diseases & Department of Cariology and Endodontics & Department of Oral Pathology, West China Hospital of Stomatology, Sichuan University, Chengdu 610041, China; ^2^Department of Operative Dentistry and Endodontics, West China School of Stomatology, Sichuan University, Chengdu 610041, China; ^3^Stomatology Center, The First Affiliated Hospital of Zhengzhou University, Zhengzhou, 450052 Henan, China; ^4^Department of Endodontics, Periodontics and Prosthodontics, University of Maryland Dental School, Baltimore, MD 21201, USA

## Abstract

Quaternary ammonium methacrylates (QAMs) are useful antimicrobial compounds against oral bacteria. Here, we investigated the effects of two QAMs, dimethylaminododecyl methacrylate (DMADDM) and dimethylaminohexadecyl methacrylate (DMAHDM), on biofilm formation, survival and development of tolerance by biofilm, and survival and development of tolerance against QAMs after prolonged starvation. *Enterococcus faecalis* (*E*. *faecalis*), *Streptococcus gordonii* (*S*. *gordonii*), *Lactobacillus acidophilus* (*L*. *acidophilus*), and *Actinomyces naeslundii* (*A*. *naeslundii*) were used. Minimum inhibitory concentration (MIC) of QAMs against multispecies biofilm was determined. Biofilm formed under sub-MIC was observed by crystal violet staining and confocal laser scanning microscopy (CLSM). Metabolic activity was assessed by 3-(4,5-dimethylthiazol-2-yl)-2,5-diphenyltetrazolium bromide (MTT) assay and lactic acid production measurement. Development of tolerance was determined by MIC values before and after exposure to QAMs or after prolonged starvation. It was found that *E*. *faecalis* and *S*. *gordonii* could survive and form biofilm under sub-MIC of QAMs. Lactic acid production from biofilms formed under sub-MIC was significantly higher than control specimens (*p* < 0.05). The exposure to sub-MIC of QAMs promoted biofilm formation, and prolonged starvation or prolonged contact with sub-MIC helped bacteria develop tolerance against killing by QAMs.

## 1. Introduction

Dental diseases, including persistent periapical infection, are biofilm-mediated infections, which have been associated with the etiology of several systemic diseases, ranging from arthritis to neurodegenerative diseases [1–4]. Biofilm is a community of bacteria enclosed in the self-produced polymeric matrix and adhered to the surface. Growth inside biofilm facilitates bacteria to survive and produce chronic infection [5]. *Enterococcus* spp., *Lactobacillus* spp., *Streptococcus* spp., and *Actinomyces* spp. are the most common species in persistent periapical infection [6, 7]. These bacteria can be well entrenched in the root canal system under lethal concentrations of root canal irrigants and starvation due to biofilm formation and synergistic effects.

Controlling bacterial infection is the key and difficult point in the prevention and treatment of endodontic infections. The use of antimicrobial compounds or antibiotics is an effective way to control bacterial infection in the root canal system [8–16]. However, due to the special structure of the root canal system, bacteria are often starved along with their changed growth metabolism and sensitivity to antibacterial drugs. So drug resistance induced by antibiotics is also a challenge for the application of antibacterial agents. The gradient of these agents decreases with the passing time, and it reaches the concentration that could not inhibit bacterial adhesion or biofilm formation. Previous studies have shown that bacteria could make varied changes in their response to antimicrobial compounds at subminimum inhibitory concentration, such as changes in morphology, growth kinetics, coaggregation mechanism, and enzyme and toxin expression [17–21]. Some studies have shown that antimicrobial compounds under sub-MIC could reduce biofilm formation while some may influence biofilm formation on the opposite side [17, 18, 21].

Quaternary ammonium methacrylates (QAMs) are one kind of cationic compounds with promising antibacterial and antibiofilm action against oral bacteria [22–24]. They can be copolymerized and covalently bonded in the dental materials to form polymer matrices. The QAMs, immobilized material surfaces, are highly positively charged, which can attract the negatively charged bacteria. Furthermore, the bacterial membrane would be penetrated and interrupted by the long fatty alkyl chains of QAMs. This unique “contact killing” antibacterial mechanism made QAMs provide long-term contact inhibition against biofilm [25–28]. In our preliminary study, two kinds of novel QAMs have been synthesized, dimethylaminododecyl methacrylate (DMADDM) and dimethylaminohexadecyl methacrylate (DMAHDM), with strong antibiofilm potency [24–32]. And they have been incorporated into dental materials and cross-linked with the resin matrix to form nonreleasing antimicrobial materials. Recently, it was found that monomers of these QAMs could get adsorbed inside the dentine block and inhibit bacterial colonization on its surface [22] so that these compounds could achieve subminimum inhibitory concentrations. But by now, no study has been reported about their tolerance against killing after exposure to prolonged starvation and biofilm formation ability under sub-MIC of antibacterial compounds. Consequently, a better understanding of the response of oral biofilm to these sub-MIC of QAMs in the oral cavity would allow clinicians to make better informed choices of antibacterial agents.

Therefore, the objectives of this study were to investigate for the first time the following: (1) the survival status and drug resistance of bacteria in the infected root canal exposed to prolonged starvation and (2) the growth and cross-resistance of bacterial biofilms under the sub-MIC of DMADDM and DMAHDM.

## 2. Materials and Methods

### 2.1. Synthesis of Antimicrobial Compounds

DMADDM was obtained after chemical reaction between tertiary ammonium compound 2-(dimethylamino)ethyl methacrylate (DMAEMA) (Sigma-Aldrich) and respective organo-halide by modified Menschutkin reaction [33]. Briefly, 10 mmol of DMAEMA, 10 mmol of 1-bromododecane (BDD), and 3 g ethanol were mixed together, capped, and starred at 70°C for 24 h in a vial. After 24 h, the vial was left open for ethanol to evaporate, which left a clear viscous liquid of DMADDM behind [34]. Likely, DMAHDM was synthesized via modified Menschutkin reaction where a tertiary amine group was reacted with an organo-halide [35].

### 2.2. Bacterial Selection and Incubation

*Enterococcus faecalis* (*E*. *faecalis*, ATCC 19433), *Streptococcus gordonii* (*S*. *gordonii*, ATCC 10558), *Lactobacillus acidophilus* (*L*. *acidophilus*, ATCC 4356), and *Actinomyces naeslundii* (*A*. *naeslundii*, ATCC 12104) were obtained from State Key Laboratory of Oral Diseases (West China School of Stomatology, Sichuan University, China). Bacteria were incubated individually overnight anaerobically (90% N_2_, 5% CO_2_, and 5% H_2_) at 37°C in brain-heart infusion (BHI) broth (Sigma-Aldrich). 10 *μ*L of overnight grown bacterial suspension was transferred to fresh BHI broth and incubated as described before. But for the starvation test, bacteria were cultured in distilled water, without the supply of glucose or other nutrients in media.

### 2.3. Minimum Inhibitory Concentration (MIC) and Minimum Bactericidal Concentration (MBC)

To evaluate the antibacterial properties of DMADDM and DMAHDM, a microtiter plate assay was used to determine the MIC and MBC for the four species. The MIC was measured as the lowest concentration of an antimicrobial agent at which no visible bacterial growth appeared, while MBC was the minimal concentration that produced no bacterial growth on an agar plate. MIC measurements were conducted according to the serial twofold microdilution method using BHI broth [36, 37]. DMADDM and DMAHDM were dissolved in sterile distilled water at 200 *μ*g/mL and diluted with a culture medium to prepare the starting concentrations.

### 2.4. MTT Assay

3-(4,5-Dimethylthiazol-2-yl)-2,5-diphenyltetrazolium bromide (MTT) could be reduced by enzymes in viable cells to form purple color formazan, thus reflecting the metabolic activity of the biofilms. Briefly, 1 mL of MTT dye (0.5 mg/mL in PBS) was added to each well and incubated anaerobically at 37°C for 1 h. After 1 h, the disk was transferred to a new 12-well plate and 1 mL of dimethyl sulfoxide (DMSO) was added to solubilize formazan crystals. After brief mixing, DMSO solution was then transferred into a 96-well plate and the absorbance was read at 540 nm (OD_540_) [26]. Three replicates were tested for each group.

### 2.5. Lactic Acid Production Measurement

In order to investigate the lactic acid production by biofilm, disks with 5- to 30-day biofilms were washed twice with PBS, then immersed in 1.5 mL buffered peptone water (BPW) (Sigma-Aldrich) supplemented with 0.2% sucrose and incubated at 37°C in 5% CO_2_ for 3 h. The lactate concentrations in BPW were determined using a lactate dehydrogenase enzymatic method by measuring OD_340nm_ [29]. Three replicates were tested for each group.

### 2.6. DNA Isolation and Real-Time Polymerase Chain Reaction

To monitor bacterial composition shifts in biofilm, DNA isolation and quantitative real-time polymerase chain reaction (qPCR) were performed. Total DNA of biofilms was isolated and purified using a TIANamp Bacteria DNA kit (TIANGEN, Beijing, China) according to the manufacturer's instructions. The bacteria were lysed using the enzymatic lysis buffer (20 mM Tris-HCl, pH 8.0; 2 mM sodium EDTA; and 1.2% Triton X-100) containing 25 mg/mL of lysozyme at 37°C for 1.5 h. The purity and concentration of DNA were detected by a NanoDrop spectrophotometer (Thermo Fisher Scientific, USA). The extracts were stored at -20°C for later use [24].

The qPCR was performed by a Bio-Rad CFX96™ Real-Time System (Bio-Rad, CA, USA). Each real-time PCR reaction mix consisting of 10 *μ*L of TaqMan Universal PCR Premix Ex Taq, 1.5 *μ*L of template, 250 nM (each) of sense and antisense primers, and 250 nM of TaqMan probes was placed into each well, and the cycling conditions used are as follows: 95°C for 3 min, followed by 40 cycles of 95°C for 10 s and 56°C for 30 s. Fluorescence was detected after each cycle. The specificity of probes was confirmed by conventional PCR, and the standard curves of these bacteria were plotted for each primer/probe set by using threshold cycle values obtained by amplifying successive 10-fold dilutions of known concentrations of DNA, which stands for the corresponding concentration of bacteria from 10^9^ CFUs to 10^4^ CFUs. The quantifications of three strains were calculated based on standard curves generated using respective standard strains. Three replicates were tested for each group. Primers used for the reaction are listed in Table 1.

### 2.7. Confocal Laser Scanning Microscopy Analysis

To determine the biomass of biofilm and volume of extracellular polysaccharide (EPS) in biofilm, confocal laser scanning microscopy (CLSM) analysis was performed. Briefly, biofilms on the disks were washed three times with PBS and then stained using the BacLight LIVE/DEAD bacterial viability kit (Molecular Probes, Eugene, OR, USA). The disks were examined using an inverted epifluorescence microscope (Eclipse TE2000-S; Nikon, Melville, NY, USA). An EPS assay was conducted according to a previous study [38]. In brief, the bacterial cells were labeled with 2.5 *μ*mol/L SYTO9 green fluorescent nucleic acid stain (480 nm/500 nm; Molecular Probes, Eugene, OR, USA). The polysaccharides were labeled with 2.5 *μ*mol/L Alexa Fluor 647-dextran conjugate (Thermo Fisher Scientific, Waltham, MA, USA). The disks with biofilms were examined using confocal laser scanning microscopy (Leica, Wetzlar, Germany).

### 2.8. Biofilm Cross-Resistance Analysis

To investigate the correlation between antibacterial agents, including DMADDM and DMAHDM, in bacterial cross-resistance, biofilm cross-resistance analysis was performed. The 30-day-old bacterial sample after starvation and bacterial samples from 10 days, 20 days, and 30 days of incubation under sub-MIC were collected and incubated anaerobically overnight in BHI broth at 37°C. The overnight grown bacteria were subjected to test MIC and MBC as described above. Bacteria grown under sub-MIC of DMADDM and DMAHDM were tested against the same compounds and exchanged to determine cross-reactivity to each other.

### 2.9. Statistical Analysis

Statistical analyses were performed using SPSS, version 22.0 (SPSS Inc., Chicago, IL, USA). One-way analyses of variance (ANOVA) were performed to detect the significant effects of the variables. Tukey's multiple comparison test was used to compare the means of each of the groups. The differences in the means of data were considered significant if *p* < 0.05.

## 3. Results

### 3.1. Starvation Resistance of Biofilm

**Figure 1:**
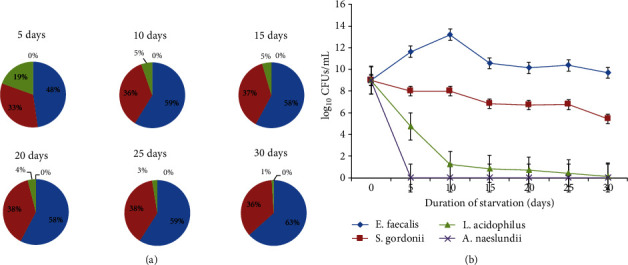
Analysis of bacterial percentage in biofilm after 30-day starvation. The results are presented as mean ± standard deviation, *n* = 3.

Figure 1(a) shows the logarithm of the number of viable bacteria after 30 days of starvation. The number of *E*. *faecalis*, *S*. *gordonii*, and *L*. *acidophilus* was significantly increased on day 15 (*p* < 0.05). The proportion of *E*. *faecalis* increased significantly between day 1 to day 10 and day 25 to day 30 (*p* < 0.05). The proportion of *S*. *gordonii* also increased on day 20 before observation, and the growth trend continued until day 25, after which it began to decline. The proportion of *L*. *acidophilus* decreased significantly on day 10 (*p* < 0.05) and decreased to 1% on day 30.

The change in the number of each bacterium is shown in Figure 1(b). *E*. *faecalis* grew fastest in the first 10 days (*p* < 0.05); then, this condition continued until day 25, after which the colony units decreased; the number of colony units significantly decreased from day 10 to day 15, but no significant change thereafter (*p* > 0.05). By the end of the observation period, the *E*. *faecalis* colony count increased by one log compared with the first day. The *S*. *gordonii* colony count was reduced by one log on day 15. The *L*. *acidophilus* colony count decreased significantly on day 10 (*p* < 0.05), and the decline continued until the end of the observation period, but the decrease was not as significant as day 10 (*p* > 0.05). Statistical analysis showed that *E*. *faecalis* was the dominant flora in the suspension, followed by *S*. *gordonii* (*p* < 0.05).

### 3.2. MIC, MBC of Bacteria

Table 2 shows the changes in tolerance of DMADDM and DMAHDM to bacteria after 30 days of starvation. The results were compared with the MIC, MBC, and obtained in the previous experiments. The MIC of DMADDM was unchanged, but the MBC and MBIC values doubled. After 30 days of starvation, the MIC and MBC of DMAHDM increased by twofold.

### 3.3. MTT Assay and Lactic Acid Production Measurement

**Figure 2:**
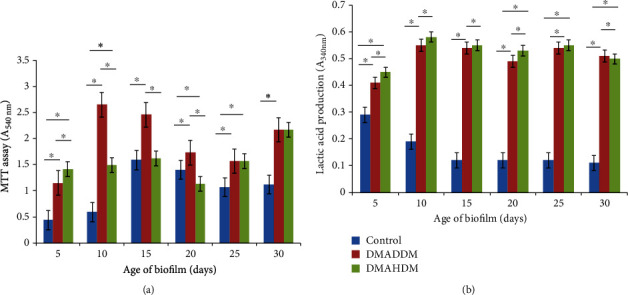
MTT assay and lactic acid production measurement. (a) Absorbance values of biofilm exposed to sub-MIC of QAMs by MTT assay. (b) Absorbance values of biofilm exposed to sub-MIC of QAMs by lactic acid production measurement. The results are expressed as mean ± standard deviation, *n* = 3. ^∗^*p* < 0.05.

As shown in Figure 2(a), the metabolic activity of biofilm in the control group increased significantly on day 15 (*p* < 0.05), and then the metabolic activity did not change significantly with mature biofilm (*p* > 0.05). The biofilm bacterial metabolic activity formed under sub-MIC of DMADDM increased in the first 10 days (*p* < 0.05), significantly decreased from 10 to 25 days (*p* < 0.05), and increased again after 30 days (*p* < 0.05). The biofilm formation under sub-MIC of DMAHDM was not significantly changed in the first 15 days (*p* > 0.05), and it decreased significantly after 20 days and then gradually increased (*p* < 0.05).

As shown in Figure 2(b), the lactic acid production of the control group showed a downward trend (*p* < 0.05). Under sub-MIC of DMADDM, lactic acid production gradually increased with biofilm ripening (*p* < 0.05). There was a decrease on day 20 and day 30 (*p* < 0.05). Similarly, the amount of lactic acid on day 10 increased (*p* < 0.05) and then gradually decreased (*p* < 0.05). Compared with the lactic acid production in the two experimental groups, it was found that biofilm produced by the DMAHDM group had more lactic acid, and the difference was statistically significant except day 25 (*p* < 0.05).

### 3.4. Analysis of Individual Bacteria in Biofilm by qPCR

**Figure 3:**
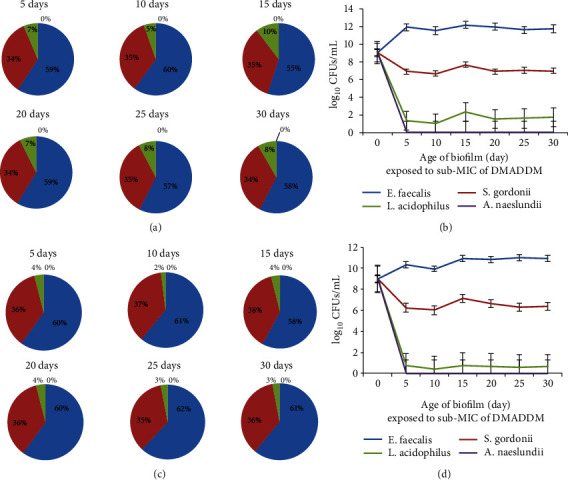
Analysis of bacterial percentage in biofilm exposed to sub-MIC of QAMs. (a) The bacterial percentage in biofilm under sub-MIC of DMADDM. (b) Colony count of each bacterial species under sub-MIC of DMADDM. (c) The bacterial percentage in biofilm under sub-MIC of DMAHDM. (d) Colony count of each bacterial species under sub-MIC of DMAHDM.

In the biofilm formed by sub-MIC of DMADDM and DMAHDM, the number of *E*. *faecalis* increased, and the numbers of *L*. *acidophilus* and *A*. *naeslundii* decreased significantly. The number of *S*. *gordonii* decreased in the first 5 days and remained unchanged afterward. The changes in the number of single species are shown in Figure 3.

### 3.5. Analysis of Biofilm Biomass and EPS Production

**Figure 4:**
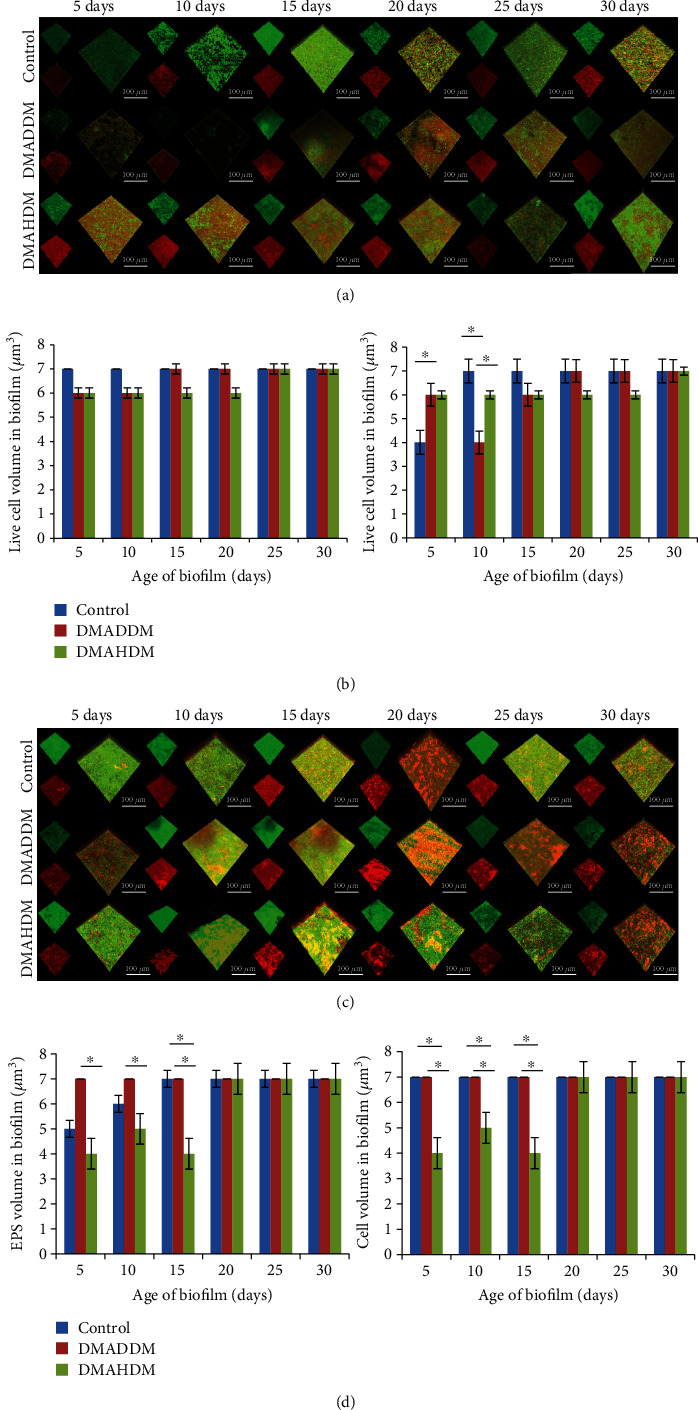
Analysis of biofilm biomass and EPS production in biofilm exposed to sub-MIC of QAMs. (a) Three-dimensional reconstruction images of biofilm under sub-MIC of DMADDM (live bacteria, stained green; dead cells, stained red). (b) Biomass analysis of live/dead bacteria under sub-MIC of DMADDM. (c) Three-dimensional reconstruction of EPS under sub-MIC of DMAHDM (bacteria, stained green; EPS, stained red). (d) Analysis of EPS production and biofilm volume under sub-MIC of DMAHDM. Data are presented as mean ± standard deviation, *n* = 3. ^∗^*p* < 0.05.

The biomass analysis of live and dead bacteria in biofilm exposed to drugs at sub-MIC is shown in Figure 4(a). There was no significant difference in the biomass of live bacteria in the control group and two experimental groups (*p* > 0.05), while the biomass of dead bacteria was less on day 5 and increased on day 10. The biofilm-producing biomass reached the peak on day 15 under sub-MIC of DMADDM, while the peak of the DMAHDM group appeared on day 25. Biomass in the two experimental groups was basically at the same level on day 5, but the biomass of dead bacteria in the DMADDM group was significantly reduced on day 10 (*p* < 0.05) and significantly increased on day 15 (*p* < 0.05). After day 20, there was no significant change in the biomass of dead bacteria.

The three-dimensional construction of EPS staining of each group is shown in Figure 4(b). In the control group, the amount of EPS production increased from day 15, reached the highest level on day 20, and remained unchanged thereafter. There was no significant change in the EPS amount under the sub-MIC of DMADDM. The biofilm formed under sub-MIC of DMAHDM had significantly lower EPS than the DMADDM group (*p* < 0.05), and it increased slightly on day 10, significantly decreased on day 15 (*p* < 0.05), and increased on day 20 (*p* < 0.05), and it remained unchanged by the end of the experiment. There was no significant difference in EPS production between the control group and the DMAHDM group (*p* > 0.05), but the EPS production of the control group reached the peak on day 15.

### 3.6. Biofilm Cross-Resistance Analysis

Table 3 shows that the biofilm formed under sub-MIC of DMADDM showed no resistance to DMADDM. However, the 10-day and 20-day biofilms doubled the MIC and MBC of DMAHDM, while the 30-day biofilm increased by 3 and 7 times, respectively. Biofilm was formed after 10 and 20 days under sub-MIC of DMAHDM, the MIC of DMAHDM increased by 8 times of the original MIC, the MBC increased by 16 times, and the MIC and MBC were at the original level on day 30.

## 4. Discussion

Quaternary ammonium methacrylates (QAMs) are a class of cationic compounds with a broad spectrum of antimicrobial effects and are widely used in consumer products for disinfection purposes. Compared to release-based biomaterials, QAMs can be copolymerized with the resin matrix to anchor into the polymer network with prolonged antimicrobial ability. QAMs are frequently detected in aquatic systems at sub-MIC and were found to affect the development of antimicrobial resistance if bacteria are exposed to increasing concentration [39, 40]. However, the effect of novel DMADDM and DMAHDM exposure to constant sub-MIC on the development of bacterial resistance in dental clinical application is unknown. In the study, we found that *E*. *faecalis* along with *S*. *gordonii* could survive and form biofilm under sub-MIC of antimicrobial compounds. Bacteria showed increased tolerance against antimicrobial compounds under short exposure to sub-MIC. Matrix production prevented bacteria from killing by antimicrobial compounds, and bacteria exposed to sub-MIC had more matrix production than bacteria growing in normal conditions in the absence of antimicrobial compounds. Therefore, a better understanding of the differential effects of various QAMs on oral biofilm could provide valuable information to help clinicians choose the best QAMs for clinical use.

QAMs affected the cell membrane and biofilm matrix. It had been suggested that the matrix of biofilms could be responsible for the increased resistance to antibiotics by acting as a diffusion barrier [41, 42]. Changes in the biofilm matrix could therefore influence the susceptibility of biofilm cells to antibiotics. Exopolysaccharides (EPS) could affect the diffusion of substances in and out of the biofilm, perhaps helping create a diverse range of microenvironments within the biofilm [43]. CLSM analysis was used in the study to identify the live/dead cell volume and cell/EPS volume in biofilm formed under sub-MIC. The analysis showed that biofilm formed under sub-MIC was significantly thinner, which indicated that biofilm formation was inhibited in the presence of antimicrobial compounds. However, the live cell volume and EPS volume from biofilm formed under sub-MIC reached a similar level with the control two to three weeks later, which indicated that surviving bacteria were capable of multiplying inside biofilm, although biofilm formation was suppressed by the presence of antimicrobial compounds. It was suggested that EPS production by bacteria under the influence of QAMs at sub-MIC also played a critical role in bacterial survival. Firstly, bacteria produced enough volume of EPS, and afterward, bacteria started multiplying to reach the maximum cell volume, but this phenomenon was not seen with biofilm formation without an antimicrobial agent. The live cell volume in the control group was at the maximum volume on its initial five days and remained constant, and EPS reached its maximum volume after two weeks. In inverse, bacteria growing under sub-MIC produced enough EPS at the beginning and cell division started after the completion of EPS production. Therefore, it was possible that QAMs at sub-MIC enhanced biofilm formation and matrix production under the influence of antimicrobial compounds that protected bacteria from the lethal effect of compounds.

Previous studies showed that after multiple exposures to DMADDM, bacteria did not develop resistance against it [36, 44]. In agreement with that, biofilm exposed to sub-MIC of DMADDM did not show any change in MIC and MBC, but the same biofilm developed tolerance against a more potent compound, DMAHDM. Similarly, biofilm under sub-MIC of DMAHDM showed increased tolerance towards DMADDM and DMAHDM, but the phenomenon was seen after 10 and 20 days of exposure while values remained constant with prolonged exposure. This suggested that there may be a clinical advantage to use DMADDM at low concentration. It would also be interesting to analyze a combination of DMADDM and DMAHDM since it was often the case that dual therapy was used clinically.

Some bacterial species, e.g., *Pseudomonas aeruginosa* isolates, were found to be resistant to QAMs. The intrinsic resistance of this organism seemed to be due to the cell wall and the cell membrane; for example, antimicrobials could not easily access their sites of action because of the lower level of permeability of the outer membrane [45], and efflux pumps caused enhanced levels of efflux of antimicrobials [46]. The cell wall was the site of action of QAMs, and bacteria persisting under sub-MIC had an active pump to push out QAMs out of the cell before getting incorporated in the cell wall [47]. The confirmed reason for the susceptibility of bacteria from mature biofilm was unknown, but we could assume that as biofilm matured, the diffusion of QAMs inside biofilm became less, which might make bacteria shut down the efflux pump and susceptible to compounds. It was suggested that constant exposure to an antimicrobial compound made bacteria tolerant against it and the development of tolerance was the first step for the development of resistance against it. Secondly, more potent and stronger compound exposure initiated the development of tolerance rapidly than their least potent compounds.

It was known that chemomechanical preparation and/or intracanal medication could significantly reduce bacteria from the root canal but could not eliminate bacteria from the root canal completely [48, 49]. It was believed that adequate sealing of the root canal would starve bacteria to death, but failed posttreatment cases showed that bacteria had survived starvation [50, 51]. So, we further identified bacteria that could survive from starvation. The qPCR analysis showed that *E*. *faecalis* and *S*. *gordonii* were common isolates after prolonged starvation and from samples of biofilm formed under sub-MIC of QAMs. The mechanism of survival of these two bacteria together was unknown, but it was proved that they could survive together after the treatment of antimicrobial compounds. The phenomenon that *E*. *faecalis* and *S*. *gordonii* could survive coincided with our previous finding where it was seen that *E*. *faecalis* and *S*. *gordonii* were common isolates and they could survive together [52]. The increase in antimicrobial resistance may compromise the use of QAMs at sub-MIC to prevent endodontic biofilm formation. New approaches are expected to be developed to eliminate biofilms from root canals and stop the spread of resistance.

## 5. Conclusion

*S*. *mutans* and *E*. *faecalis* produced resistance to antimicrobial compounds after prolonged starvation. DMADDM and DMAHDM at sub-MIC could inhibit the growth of multispecies biofilm, increase the level of metabolism, enhance the ability of acid production, and produce cross-resistance.

## Figures and Tables

**Table 1:** Bacteria-specific primers used for qPCR.

Bacteria	Primer pairs
*E*. *faecalis*	F: 5′-CGCGAACATTTGATGTGGCT-3′
	R: 5′-GTTGATCCGTCCGCTTGGTA-3′
*S*. *gordonii*	F: 5′-GCCTTAATAGCACCGCCACT-3′
	R: 5′-CCATCTCTGTTGTTAGGGCGT-3′
*L*. *acidophilus*	F: 5′-AGAGGTAGTAACTGGCCTTTA-3′
	R: 5′-GCGGAAACCTCCCAACA-3′
*A*. *naeslundii*	F: 5′-CTCCTACGGGAGGCAGCAG-3′
	R: 5′-CACCCACAAACGAGGCAG-3′

**Table 2:** Development of tolerance against DMADDM and DMAHDM after 30-day starvation.

Antimicrobial compounds	MIC (*μ*g/mL)		MBC (*μ*g/mL)	
	Control	Test	Control	Test
DMADDM	50	50	100	200
DMAHDM	12.5	25	12.5	50

**(a):** 

Antimicrobial compounds	MIC (*μ*g/mL)	MBC (*μ*g/mL)
DMADDM	50	100
DMAHDM	12.5	12.5

MIC and MBC values of biofilm exposed to DMADDM/DMAHDM.

**(b):** 

		Exposed to sub-MIC of DMADDM			Exposed to sub-MIC of DMAHDM		
		10 d	20 d	30 d	10 d	20 d	30 d
MIC (*μ*g/mL)	DMADDM	50	50	50	100	50	50
	DMAHDM	25	25	50	100	100	25

MIC values of biofilm after exposed to sub-MIC of DMADDM/DMAHDM.

**(c):** 

		Exposed to sub-MIC of DMADDM			Exposed to sub-MIC of DMAHDM		
		10 d	20 d	30 d	10 d	20 d	30 d
MBC (*μ*g/mL)	DMADDM	100	100	100	200	100	100
	DMAHDM	50	50	100	200	200	50

MBC values of biofilm after exposed to sub-MIC of DMADDM/DMAHDM.

## Data Availability

The data used to support the findings of this study are available from the corresponding authors upon request.

## References

[1] Kumar P. S. (2013). Oral microbiota and systemic disease. *Anaerobe*.

[2] Frédéric L., Michel B., Selena T. (2018). Oral microbes, biofilms and their role in periodontal and peri-implant diseases. *Materials*.

[3] Fiorillo L. (2020). We do not eat alone: formation and maturation of the oral microbiota. *Biology*.

[4] Fiorillo L., Cervino G., Laino L. (2019). Porphyromonas gingivalis, periodontal and systemic implications: a systematic review. *Dentistry Journal*.

[5] Lewis K. (2008). Multidrug tolerance of biofilms and persister cells. *Current Topics in Microbiology and Immunology*.

[6] Bouillaguet S., Manoil D., Girard M. (2018). Root microbiota in primary and secondary apical periodontitis. *Frontiers in Microbiology*.

[7] Antunes H. S., Rôças I. N., Alves F. R. F., Siqueira J. F. (2015). Total and specific bacterial levels in the apical root canal system of teeth with post-treatment apical periodontitis. *Journal of Endodontics*.

[8] Nakata T., Kitasako Y., Sadr A., Nakashima S., Tagami J. (2018). Effect of a calcium phosphate and fluoride paste on prevention of enamel demineralization. *Dental Materials Journal*.

[9] Cummins D. (2013). The development and validation of a new technology, based upon 1.5% arginine, an insoluble calcium compound and fluoride, for everyday use in the prevention and treatment of dental caries. *Journal of Dentistry*.

[10] da Costa L. F. N. P., da Silva Furtado Amaral C., da Silva Barbirato D., Leão A. T. T., Fogacci M. F. (2017). Chlorhexidine mouthwash as an adjunct to mechanical therapy in chronic periodontitis: a meta-analysis. *Journal of American Dental Association*.

[11] Pithon M. M., Sant’Anna L. I. D. A., Baião F. C. S., dos Santos R. L., da Silva Coqueiro R., Maia L. C. (2015). Assessment of the effectiveness of mouthwashes in reducing cariogenic biofilm in orthodontic patients: a systematic review. *Journal of Dentistry*.

[12] Gonçalves L. S., Rodrigues R. C. V., Junior C. V. A., Soares R. G., Vettore M. V. (2016). The effect of sodium hypochlorite and chlorhexidine as irrigant solutions for root canal disinfection: a systematic review of clinical trials. *Journal of Endodontics*.

[13] Mohammadi Z., Jafarzadeh H., Shalavi S. (2014). Antimicrobial efficacy of chlorhexidine as a root canal irrigant: a literature review. *Journal of Oral Science*.

[14] Haapasalo M., Shen Y., Wang Z., Gao Y. (2014). Irrigation in endodontics. *British Dental Journal*.

[15] Zhang J. F., Wu R., Fan Y. (2014). Antibacterial dental composites with chlorhexidine and mesoporous silica. *Journal of Dental Research*.

[16] Meto A., Colombari B., Sala A. (2019). Antimicrobial and antibiofilm efficacy of a copper/calcium hydroxide-based endodontic paste against *Staphylococcus aureus, Pseudomonas aeruginosa and Candida albicans*. *Dental Materials Journal*.

[17] Wright E. A., Fothergill J. L., Paterson S., Brockhurst M. A., Winstanley C. (2013). Sub-inhibitory concentrations of some antibiotics can drive diversification of *Pseudomonas aeruginosa* populations in artificial sputum medium. *BMC Microbiology*.

[18] Bedran T. B. L., Grignon L., Spolidorio D. P., Grenier D. (2014). Subinhibitory concentrations of triclosan promote Streptococcus mutans biofilm formation and adherence to oral epithelial cells. *PLoS One*.

[19] Andreoni F., Zürcher C., Tarnutzer A. (2017). Clindamycin affects group a *streptococcus* virulence factors and improves clinical outcome. *Journal of Infectious Diseases*.

[20] Gemmell C. G., Ford C. W. (2002). Virulence factor expression by Gram-positive cocci exposed to subinhibitory concentrations of linezolid. *Journal of Antimicrobial Chemotherapy*.

[21] Wojnicz D., Tichaczek-Goska D. (2013). Effect of sub-minimum inhibitory concentrations of ciprofloxacin, amikacin and colistin on biofilm formation and virulence factors of *Escherichia coli* planktonic and biofilm forms isolated from human urine. *Brazilian Journal of Microbiology*.

[22] Chen H., Han Q., Zhou X. (2017). Heat-polymerized resin containing dimethylaminododecyl methacrylate inhibits Candida albicans biofilm. *Materials*.

[23] Cheng L., Zhang K., Zhang N. (2017). Developing a new generation of antimicrobial and bioactive dental resins. *Journal of Dental Research*.

[24] Wang H., Wang S., Cheng L. (2019). Novel dental composite with capability to suppress cariogenic species and promote non-cariogenic species in oral biofilms. *Materials Science and Engineering: C*.

[25] Cheng L., Weir M. D., Xu H. H. K. (2012). Antibacterial amorphous calcium phosphate nanocomposites with a quaternary ammonium dimethacrylate and silver nanoparticles. *Dental Materials*.

[26] Tiwari S. K., Wang S., Huang Y. (2020). The antibacterial effects of quaternary ammonium salts in the simulated presence of inhibitors in root canals: a preliminary in-vitro study. *Coatings*.

[27] Baras B. H., Wang S., Melo M. A. S. (2019). Novel bioactive root canal sealer with antibiofilm and remineralization properties. *Journal of Dentistry*.

[28] Fei X., Li Y., Weir M. D. (2020). Novel pit and fissure sealant containing nano-CaF_2_ and dimethylaminohexadecyl methacrylate with double benefits of fluoride release and antibacterial function. *Dental Materials*.

[29] Zhou W., Ren B., Zhou X. (2016). Novel cavity disinfectants containing quaternary ammonium monomer dimethylaminododecyl methacrylate. *Materials*.

[30] Liang J., Li M., Ren B. (2018). The anti-caries effects of dental adhesive resin influenced by the position of functional groups in quaternary ammonium monomers. *Dental Materials*.

[31] Chen H., Tang Y., Weir M. D. (2020). Effects of S. *mutans* gene-modification and antibacterial monomer dimethylaminohexadecyl methacrylate on biofilm growth and acid production. *Dental Materials*.

[32] Zhou Y., Wang S., Zhou X. (2019). Short-Time Antibacterial Effects of Dimethylaminododecyl Methacrylate on Oral Multispecies Biofilm In Vitro. *BioMed Research International*.

[33] Antonucci J. M., Zeiger D. N., Tang K., Lin-Gibson S., Fowler B. O., Lin N. J. (2012). Synthesis and characterization of dimethacrylates containing quaternary ammonium functionalities for dental applications. *Dental Materials*.

[34] Li F., Weir M. D., Xu H. H. (2013). Effects of quaternary ammonium chain length on antibacterial bonding agents. *Journal of Dental Research*.

[35] Cheng L., Weir M. D., Zhang K., Arola D. D., Zhou X., Xu H. H. K. (2013). Dental primer and adhesive containing a new antibacterial quaternary ammonium monomer dimethylaminododecyl methacrylate. *Journal of Dentistry*.

[36] Wang S., Wang H., Ren B. (2017). Do quaternary ammonium monomers induce drug resistance in cariogenic, endodontic and periodontal bacterial species?. *Dental Materials*.

[37] Adukwu E. C., Allen S. C., Phillips C. A. (2012). The anti-biofilm activity of lemongrass (*Cymbopogon flexuosus*) and grapefruit (*Citrus paradisi*) essential oils against five strains of *Staphylococcus aureus*. *Journal of Applied Microbiology*.

[38] Zheng X., Zhang K., Zhou X. (2013). Involvement of *gshAB* in the interspecies competition within oral biofilm. *Journal of Dental Research*.

[39] Tabata A., Nagamune H., Maeda T., Murakami K., Miyake Y., Kourai H. (2003). Correlation between resistance of Pseudomonas aeruginosa to quaternary ammonium compounds and expression of outer membrane protein OprR. *Antimicrobial Agent and Chemotherapy*.

[40] Voumard M., Venturelli L., Borgatta M. (2020). Adaptation of Pseudomonas aeruginosa to constant sub-inhibitory concentrations of quaternary ammonium compounds. *Environmental Science: Water Research & Technology*.

[41] Beer D., Srinivasan D. R., Stewart P. S. (1994). Direct measurement of chlorine penetration into biofilms during disinfection. *Applied and Environmental Microbiology*.

[42] Gerke C., Kraft A., Sussmuth R., Schweitzer O., Gotz F. (1998). Characterization of the N-acetylglucosaminyltransferase activity involved in the biosynthesis of the Staphylococcus epidermidis polysaccharide intercellular adhesin. *The Journal of Biological Chemistry*.

[43] Koo H., Falsetta M. L., Klein M. I. (2013). The exopolysaccharide matrix. *Journal of Dental Research*.

[44] Wang S., Wang H., Ren B. (2018). Drug resistance of oral bacteria to new antibacterial dental monomer dimethylaminohexadecyl methacrylate. *Scientific Reports*.

[45] Nikaido H. (1994). Prevention of drug access to bacterial targets: permeability barriers and active efflux. *Science*.

[46] Li X., Nikaido H., Poole K. (1995). Role of MexA-MexB-OprM in antibiotic efflux in Pseudomonas aeruginosa. *Antimicrobial Agents and Chemotherapy*.

[47] Pu Y., Ke Y., Bai F. (2017). Active efflux in dormant bacterial cells - new insights into antibiotic persistence. *Drug Resistance Updates*.

[48] Shuping G. B., Orstavik D., Sigurdsson A., Trope M. (2000). Reduction of intracanal bacteria using nickel-titanium rotary instrumentation and various medications. *Journal of Endodontics*.

[49] Siqueira J. F., Guimarães-Pinto T., Rôças I. N. (2007). Effects of chemomechanical preparation with 2.5% sodium hypochlorite and intracanal medication with calcium hydroxide on cultivable bacteria in infected root canals. *Journal of Endodontics*.

[50] Gomes B. P. F. A., Pinheiro E. T., Gade-Neto C. R. (2004). Microbiological examination of infected dental root canals. *Oral Microbiology Immunology*.

[51] Tennert C., Fuhrmann M., Wittmer A. (2014). New bacterial composition in primary and persistent/secondary endodontic infections with respect to clinical and radiographic findings. *Journal of Endodontics*.

[52] Tiwari S. K., Guo X., Huang Y. (2019). The inhibitory effect of quaternary ammonium salt on bacteria in root canal. *Scientific Reports*.

